# SARS Preparedness and Response Planning

**DOI:** 10.3201/eid1002.030803

**Published:** 2004-02

**Authors:** Umesh D. Parashar, Larry J. Anderson

**Affiliations:** * Centers for Disease Control and Prevention, Atlanta Georgia, USA

On July 5, 2003, less than 4 months after the first cases of severe acute respiratory syndrome (SARS) were recognized, the World Health Organization (WHO) declared that the global epidemic had been contained. Although the United States was not as severely affected by the SARS epidemic as parts of Asia and Canada, the outbreak response demonstrated both known and unexpected strengths and weaknesses in U.S. national, state, and local public health and healthcare capacities to address major infectious disease challenges. Although whether SARS will reappear is unknown, the public health and healthcare communities must be prepared for the possibility. As part of the preparedness and response planning process, the Centers for Disease Control and Prevention (CDC) convened a meeting August 12–13, 2003, in Atlanta.

The meeting had approximately 100 participants, including 30 external partners from international, national, state, and local agencies. The purpose of the meeting was to share experiences and lessons learned from the response to the SARS outbreak, describe anticipated needs in preparation for the possible reemergence of SARS, discuss SARS preparedness and response plans currently under development, and outline priority areas and roles of various partners in ensuring adequate preparedness at the national, state, and local level.

Two plenary sessions and a breakout session were held. The speakers in the first plenary outlined several key lessons learned during the outbreak response: 1) although some clinical features are suggestive of SARS, its symptoms overlap too much with those of other respiratory pathogens to make a clinical diagnosis; 2) risk of exposure is key to considering the likelihood of a diagnosis of SARS; 3) prompt use of isolation and infection control procedures was a key and effective part of SARS control; 4) quarantine was an integral part of SARS control in some settings with extensive transmission; and 5) testing multiple specimens (e.g., respiratory secretions, stool, and serum or plasma) may improve our ability to detect SARS-associated coronavirus (SARS-CoV) infection.

The speakers also described U.S. national, state, and local perspectives on SARS preparedness planning. They emphasized the need to integrate SARS preparedness planning with other preparedness efforts, such as those for pandemic influenza and bioterrorism, and to address legal, policy, and authority issues in responding to public health emergencies like SARS. The importance of international collaboration and cooperation in responding to an outbreak such as SARS and preparing for its possible return was also emphasized.

The speakers in the second plenary session highlighted the following lessons learned at the federal, provincial, and local levels during the SARS outbreak in Toronto, Canada: 1) public health units need flexible and robust surveillance and information technology systems to handle data-collection needs and facilitate rapid reporting of disease activity across and within multiple jurisdictions; 2) isolation and quarantine measures are acceptable if appropriately explained, but it is important to address issues of identification and tracking of contacts, to monitor potential contacts for noncompliance, and to provide them with social and economic support; 3) public health programs and hospitals require extensive expertise, resources, and good training to strengthen infection control practices; 4) laboratories should develop standard protocols and agreements regarding specimen and data sharing and ownership; and 5) accurate and timely dissemination of information are critical and should be tailored to the needs of specific groups, be easily accessible, and be culturally and linguistically appropriate.

Following the first plenary session, participants were divided into five workgroups to cover the following components of the SARS preparedness and response plans: 1) surveillance and information technology; 2) community preparedness and response (including isolation and quarantine); 3) healthcare preparedness; 4) laboratory; and 5) communications and education. Each workgroup was asked to define the key issues or needs for an effective response to SARS, preparedness activities that should be begun immediately, and the roles of federal, state, and local agencies and hospitals in these efforts. During the second plenary, each workgroup presented a summary of their discussions to the larger group of participants.

For surveillance, a flexible and functional response plan is needed that could be adapted to the various stages of a SARS epidemic and that integrates infection control activities both within hospitals and in the community. Key preparedness activities include educating healthcare workers about the diagnosis of SARS and developing guidelines for identification, reporting, and laboratory evaluation of potential SARS case-patients. Establishing an efficient data management system that links clinical, epidemiologic, and laboratory data and allows rapid sharing of critical and pertinent information was identified as a high priority.

For community response, guidelines should address issues of isolation and quarantine of SARS patients and their contacts, including consideration of facilities for isolation (hospital, residential, other) and mechanisms of enforcement. The guidelines should be flexible and allow state or local officials to use their knowledge of local circumstances and judgment to determine which measures are most applicable. Successful implementation of containment measures will depend on public trust and require a consistent and clear communications plan. Groups that will be instrumental in implementing an effective response, such as the transportation industry, law enforcement, emergency services, and federal, state, and local legal experts, should be engaged early in the planning process. Training modules and drills that utilize realistic scenarios to evaluate the decision-making process and assess the feasibility of implementing containment measures should be developed, tested, and disseminated.

For healthcare preparedness, key considerations include defining infection control precautions for evaluating and handling patients with respiratory illness in the outpatient and inpatient setting, educating and training clinicians on clinical features of SARS and appropriate use of personal protective equipment, and building strong partnerships and collaborations between the clinical and public health communities, including cross-training staff in the areas of infection control and public health. Furthermore, issues of resource allocation and surge capacity in the event of a major SARS epidemic should be addressed.

For laboratory preparedness, guidelines should be updated for specimen collection, transport, and storage and the appropriate use of diagnostic tests and interpretation of test results. Surge capacity for testing at the federal, state, and local levels should be identified, and an adequate supply of reagents that have been properly validated and checked for quality should be ensured. While research to develop second-generation assays for improved diagnosis of SARS-CoV infection should continue, efforts should also focus on improving the performance of existing assays. Biosafety recommendations for specimen collection and laboratory processing must be updated. Guidelines for environmental testing for SARS-CoV must be developed and should include information on the role and utility of testing.

For communications and education, messages and curricula should be developed that target three audiences: public (including policy makers), physicians, and public health workers. Materials that were developed in response to the SARS outbreak must be reviewed and updated. Education and training efforts should focus on key areas, such as recognizing the clinical manifestations of SARS, appropriate use of infection control practices and personal protective equipment, rationale and practical guidance for implementing isolation and quarantine, and appropriate use and interpretation of laboratory diagnostic tests.

The information and ideas shared in this meeting are helping the public health and healthcare communities define priority SARS preparedness activities at the national, state, and local levels.

